# *Aurka* loss in CD19^+^ B cells promotes megakaryocytopoiesis via IL-6/STAT3 signaling-mediated thrombopoietin production

**DOI:** 10.7150/thno.49007

**Published:** 2021-03-04

**Authors:** Xin Chen, Chennan Wang, Na Sun, Shuai Pan, Rongqing Li, Xueqin Li, Jie Zhao, Huan Tong, Yangyang Tang, Jing Han, Jianlin Qiao, Hongbin Qiu, Hui Wang, Jing Yang, Takayuki Ikezoe

**Affiliations:** 1 Jiangsu Province Key Laboratory of Immunity and Metabolism, Xuzhou Medical University, Xuzhou, Jiangsu, China.; 2 Department of Pathogenic Biology and Immunology, Xuzhou Medical University, Xuzhou, Jiangsu, China.; 3 School of Basic Medicine, Jiamusi University, Jiamusi, Heilongjiang, China.; 4 Department of Hematology, The First Affiliated Hospital of Xuzhou Medical University, Xuzhou, Jiangsu, China.; 5 National Experimental Demonstration Center for Basic Medicine Education, Xuzhou Medical University, Xuzhou, Jiangsu, China.; 6 The Department of Hematology, Fukushima Medical University, Fukushima, Japan.

**Keywords:** Aurora-A, thrombopoietin, STAT3, IL-6, megakaryocytopoiesis.

## Abstract

**Rationale**: Aurora kinase A (Aurora-A), which is required for mitosis, is a therapeutic target in various tumors. Targeting Aurora-A led to an increase in the differentiation and polyploidization of megakaryocytes both *in vivo* and *in vitro*. However, the mechanisms involved in controlling megakaryocyte differentiation have not been fully elucidated.

**Methods:** Conditional *Aurka* knockout mice were generated. B cell development, platelet development and function were subsequently examined. Proplatelet formation, *in vivo* response to mTPO, post-transfusion experiment, colony assay, immunofluorescence staining and quantification, and ChIP assay were conducted to gain insights into the mechanisms of *Aurka* loss in megakaryocytopoiesis.

**Results:** Loss of *Aurka* in CD19^+^ B cells impaired B cell development in association with an increase in the number of platelets in peripheral blood (PB). Surprisingly, thrombopoietin (TPO) production and IL-6 were elevated in the plasma in parallel with an increase in the number of differentiated megakaryocytes in the bone marrow (BM) of *Aurka^f/f^;Cd19^Cre/+^* mice. Interestingly, compared with that of the* Aurka^f/f^* mice, a higher number of CD19^+^ B cells close to megakaryocytes was observed in the BM of the *Aurka^f/f^;Cd19^Cre/+^* mice. Moreover, *Aurka* loss in CD19^+^ B cells induced signal transducer and activator of transcription-3 (STAT3) activation. Inhibition of STAT3 reduced the *Tpo* mRNA levels. ChIP assays revealed that STAT3 bound to the TPO promoter. Additionally, STAT3-mediated TPO transcription was an autocrine effect provoked by IL-6, at least partially.

**Conclusions:** Deletion of *Aurka* in CD19^+^ B cells led to an increase in IL-6 production, promoting STAT3 activation, which in turn contributed to TPO transcription and megakaryocytopoiesis.

## Introduction

Aurora-A, one of the members of the serine/threonine kinase family, is required for centrosome maturation, mitotic spindle formation, and accurate chromosome segregation 1. Aberrant expression of Aurora-A has been observed in various types of cancers, including colorectal cancer and hematopoietic malignancies 2, 3, indicating that Aurora-A is an attractive target for antitumor therapeutic strategies. However, deletion of the *Aurora* gene resulted in early embryonic lethality 4, and it is difficult to investigate the contributions of Aurora-A to tumorigenesis under actual physiological conditions. In an inducible mouse model with hematopoietic-specific *Aurka* deletion, loss of* Aurka* led to specific enrichment of differentiated megakaryocytes 1. Additionally, alisertib, a specific inhibitor of Aurora-A, induced polyploidization and differentiation as assessed by CD41 and CD42 expression in megakaryocyte cells 5. These observations indicated that Aurora-A was dispensable for megakaryocyte polyploidization and differentiation 1. Nevertheless, the mechanisms involved in controlling megakaryocyte maturation mediated by *Aurka* deletion remain obscure.

TPO is required for the survival, proliferation and differentiation of BM megakaryocytes 6, 7. Hepatocytes are one of the major sources of TPO production and secretion 8. In a dextran sodium sulfate (DSS)-induced mouse model of colitis, the thrombocytosis response was observed in wild-type (WT) mice but not in *Il6^-/-^* mice 9. Additionally, many studies found that the proinflammatory cytokine IL-6 stimulated hepatic TPO synthesis 10, 11, indicating that IL-6 could be involved in mediating the differentiation of megakaryocytes by regulating TPO synthesis.

STAT3, a member of the STAT family, has been extensively studied for its function as a transcriptional regulator and its role as a mediator of development, normal physiology, and pathology in many diseases 12. In response to cytokines and growth factors, STAT3 is phosphorylated and activated. Activated STAT3 shuttles into the nucleus and binds to the interferon-gamma activated sequence (GAS) within target gene promoters to regulate gene transcription 13. A recent study showed that upon Jak2 inhibitors, the *Tpo* mRNA levels were mediated by the Ashwell-Morell receptor (AMR) signal in association with a decrease in the phosphorylated forms of STAT3 in HepG2 cells 14. However, the roles of STAT3 in *Aurka* loss-mediated differentiation of megakaryocytes are unknown. In this study, we found that loss of *Aurka* in CD19^+^ B cells contributed to the differentiation of megakaryocytes and platelet production via STAT3-mediated TPO transcription, at least partially.

## Materials and Methods

### Generation of the mice with conditional *Aurka* knockout

Conditional knockout of *Aurka* was generated as described previously 15. The *Cd19^Cre/+^* mice (B6.SJL-Tg(*Cd19Cre*)997Gum/J) were intercrossed with mice carrying loxP-flanked *Aurka* alleles (*Aurka^f/f^*). PCR was utilized to identify the conditional *Aurka* knockout using primers (p5 5′-GGTAAGTGGTCTTGGGTGCT-3′; p6 5′- TAGCCAACTCATCTCCTCTG-3′) and *Cd19Cre* (OIMR1084: 5′- GCGGTCTGGCAGTAAAAACTATC -3′; OIMR1085: 5′- GTGAAACAGCATTGCTGTCACTT -3′; OIMR1589: 5′- CCTCTCCCTGTCTCCTTCCT-3′; OIMR1590: 5′- TGGTCTGAGACATTGACAATCA-3′) alleles. Mice were strictly bred and maintained under protocols approved by the Institutional Animal Care and Use Committee at Xuzhou Medical University. Six- to eight-week-old age- and sex-matched mice were used for the animal experiments.

### Surface staining, flow cytometry and cell sorting

Spleen and BM cell suspensions were prepared as described 16. After being washed with PBS twice, cells were blocked with either rat anti-mouse CD16/CD32 antibody (2.4G2, BD Biosciences) or APC-Cy7-anti-CD16/CD32 (2.4G2, isotypic control (IC): APC-Cy7-IgG2b, κ, A95-1, BD Biosciences) on ice for 10 min. After which, the cells were stained with indicated antibodies with directly-conjugated fluorochromes (dilution 1/100) or IC antibodies specific to each antibody used (dilution 1/100). Data were analyzed using FlowJo (FlowJo_V10).

For B cell analysis in BM or spleens, Percp-Cy5.5-anti-CD45 (30-F11, IC: PerCP-Cy5.5-IgG2b, κ, A95-1, BD Biosciences, CA, USA), PerCP-anti-B220 (RA3-6B2, IC: PerCP-IgG2a, κ, R35-95, BD Biosciences), V450-anti-CD19 (1D3, IC: V450-IgG2a, κ, R35-95, BD Biosciences), APC-anti-CD21 (7G6, IC: APC-IgG2b, κ, A95-1, BD Biosciences), PE-anti-CD23 (B3B4, IC: PE-IgG2a, κ, R35-95, BD Biosciences), FITC-anti-CD24 (M1-69, IC: FITC-IgG2b, κ, A95-1, BD Biosciences), PE-anti-CD43 (S7, IC: PE-IgG2a, κ, R35-95, BD Biosciences), PE/Cy7-anti-IgM (R6-60.2, IC: PE/Cy7-IgG2a, κ, R35-95, BD Biosciences), and BV510-anti-IgD (11-26c.2a, IC: BV510-IgG2a, κ, R35-95, BD Biosciences) antibodies were used.

For megakaryocyte analysis in BM, an anti-lineage marker cocktail was used: FITC-anti-TER119 (Ter119, IC: FITC-IgG2b, κ, eB149/10H5, eBioscicence), FITC-anti-B220 (RA3-6B2, IC: FITC-IgG2a, κ, RTK2758, Biolegend, San Diego, CA, USA), FITC-anti-CD11b (M1/70, IC: FITC-IgG2b, κ, A95-1, BD Biosciences), FITC-anti-Gr-1 (RB6-8C5, IC: FITC-IgG2b, κ, RTK4530, Biolegend), FITC-CD11c (N418, IC: FITC-IgG, HTK888, Biolegend), FITC-anti-CD4 (RM4-5, IC: FITC-IgG2a, κ, R35-95, BD Biosciences), FITC-anti-CD8a (53-6.7, IC: FITC-IgG2a, κ, R35-95, BD Biosciences). PE/Cyanine7-anti-c-Kit (2B8, IC: PE/Cyanine7-IgG1, κ, MOPC-21, Biolegend), APC/Cyanine7-anti-Sca-1 (D7, IC: APC/Cyanine7-IgG2a, κ, RTK2758, Biolegend), PE-anti-CD34 (RAM34, IC: PE-IgG2a, κ, R35-95, BD Biosciences), PerCP/Cyanine5.5-anti-CD41 (MWReg30, Biolegend), APC-Cy7-anti-CD16/CD32 and APC-anti-Mpl (bs-10362R, IC: APC-IgG, bs-0295P-APC, Bioss, Beijing, China) antibodies were used.

Intracellular cytokine staining was performed as described previously 17. Briefly, cells were harvested, washed and surface stained prior to intracellular staining using Fixation/Permeabilization kit (BD Biosciences) following the manufacturer's instructions. The antibodies to label the following cell surface markers and cytokines were used: FITC-anti-CD11b, PE-anti-F4/80 (T45-2342, IC: PE-IgG2a, κ, R35-95, BD Biosciences), V450-anti-CD19 (1D3, IC: V450-IgG2a, κ, R35-95, BD Biosciences), APC-anti-IL-6 (MP5-20F3, IC: APC-IgG1, κ, RTK2071, Biolegend), and FITC-anti-p-STAT3 (13A3-1, IC: FITC-IgG1, κ, MOPC-21, Biolegend).

For cell sorting, cells were stained with the indicated antibodies and sorted on a FACSAria flow cytometer. The purity of the sorted cells was greater than 99%.

### Cellularity determination

Cellularity determination was performed as previously described 18. Briefly, an aliquot of BM cell suspensions was diluted in 3% acetic acid to lyse the red blood cells and subjected to cell counting. Trypan blue staining was used to assess cell viability, which was higher than 98% in all experiments.

### Mouse megakaryocytes culture

Briefly, mouse BM cells were flushed from the femur and tibia and were then sorted for Lin^-^c-Kit^+^ cells by flow cytometry after staining with anti-lineage marker cocktail and anti-c-Kit antibodies. Lin^-^c-Kit^+^ cells were grown in 10% FBS-supplemented RPMI-1640 medium with 2 mM L-glutamine, penicillin/streptomycin, and 20 ng/mL murine stem cell factor (SCF, Peprotech; Rocky Hill, NJ, USA) at 37°C under 5% CO_2_ for 2 days. The cells were then cultured in the presence of 20 ng/mL murine SCF and 200 ng/mL murine TPO (mTPO, Peprotech) for the indicated time points to obtain murine megakaryocytes as described previously 19, 20.

### Isolation of primary mouse hepatocytes

*Aurka^f/f^* mice were treated with either control diluent or 1 µg IL-6 as previously described 10. Hepatocytes were isolated from mice by two-step collagenase perfusion. Briefly, after perfusion and dissociation, cells were filtered through a 70 mm filter. Hepatocytes were further separated and purified by centrifugation at low speed, and Percoll gradient centrifugation was performed as described previously 21-23.

### Platelet counts and preparation

Platelet, mean platelet volume and blood cell counts were performed with a Sysmex XP-100 Hematologic Analyzer (Sysmex Corporation). Platelet was prepared as previously described 24.

### Reticulated platelets (RP) analysis

RP was examined as described previously 25. Briefly, 50 μL whole blood was collected into 50 μL of 0.76% sodium citrate, and incubated with PE-CD41 (MWReg30, BD Biosciences) for 10 min in the dark at room temperature. After which, platelet rich plasma (PRP) was prepared by centrifugation (180 g for 5 min at room temperature) and diluted with 4% formaldehyde. Fixed platelets were stained with thiazole orange (100 ng/mL, Retic-count, Becton Dickinson, SA) for 30 min in the dark at room temperature, and subjected to flow cytometry. A total of 5 × 10^4^ identified platelets were collected, and the percentage of RP was analyzed.

### Proplatelet formation

Proplatelet formation was performed as previously described 20. Briefly, Lin^-^c-Kit^+^ cells from mouse BM were sorted and cultured in 10% FBS-supplemented RPMI-1640 medium with 2 mM L-glutamine and penicillin/streptomycin in the presence of 200 ng/mL mTPO for 4 days.

For coculture experiments, CD19^+^ B cells from the *Aurka^f/f^;Cd19^Cre/+^* mice were sorted and cocultured with Lin^-^c-Kit^+^ hematopoietic progenitors from normal mice in 10% FBS-supplemented RPMI-1640 medium with 2 mM L-glutamine and penicillin/streptomycin in the presence of 200 ng/mL mTPO together with murine 5 ng/mL murine IL-17 (mIL-17, Peprotech; Rocky Hill, NJ, USA). CD41^+^ von Willebrand factor (vWF)^+^ (11778-1-AP, Proteintech) cells with cytoplasmic processes longer than the diameter of the cytoplasm were defined as proplatelet-forming megakaryocytes, in which vWF staining indicates platelet buds scattered throughout proplatelets 20. The number of proplatelet-forming megakaryocytes was determined by Immunofluorescence staining. For confocal microscopy, cells were fixed with 10% formalin and permeabilized with 0.25% Triton X-100. After which, cells were stained with anti-CD41 (dilution 1/200) and anti-vWF (dilution 1/50) antibodies. The nuclei were stained with 4', 6-diamidino-2-phenylindole (DAPI). The slides were imaged by laser confocal microscope.

### Tail bleeding time

Tail bleeding time was measured to examine platelet function *in vivo* as described previously 26. Briefly, a 3-mm segment of the tail tip of each mouse was cut off, and the tail was immersed in prewarmed sterile saline solution (37°C). Tail bleeding time was defined as the time taken for bleeding to stop.

### Ploidy analysis

Hoechst 33342 (10 µg/mL, MCE) was added to the medium of cultured megakaryocytes for 2 h at 37°C as described previously 27. Cells were stained with the directly coupled monoclonal antibodies PE-anti-CD41 and APC-anti-Mpl for 30 min at 4°C. Ploidy was measured in the CD41^+^Mpl^+^ cell population.

### Transmission electron microscopy

The ultrastructure of megakaryocytes was examined by transmission electron microscopy as described previously 28. Briefly, Lin^-^c-Kit^+^ cells from mouse BM were first sorted with flow cytometry. Then, the cells were grown in 10% FBS-supplemented RPMI-1640 medium with 2 mM L-glutamine and penicillin/streptomycin medium in the presence of 20 ng/mL murine SCF and 200 ng/mL murine TPO. After 4 days, the cells were centrifuged at 1500 rpm for 10 min and washed twice with phosphate-buffered saline. Then, the pellets were immersed in a fixative consisting of 2.5% glutaraldehyde and 3% paraformaldehyde and subjected to transmission electron microscope analysis (Philips Tecnai-10; Eindhoven, The Netherlands).

### *In vivo* response to mTPO

For determination of whether the *Aurka^f/f^;Cd19^Cre/+^* mice have a different sensitivity to TPO, 6-week-old mice were subcutaneously (s.c.) injected with either 0.1% bovine BSA or mTPO at a dose of 100 ng/g body weight for 3 consecutive days as described previously 29. The number of platelets was counted every other day.

### Post-transfusion experiment

A post-transfusion experiment was performed as previously described 24. Briefly, washed murine platelets labeled with 5 μg/mL calcein (BD Biosciences) were incubated at RT for 1 h and were transfused into acceptor mice through the tail (1 × 10^8^ platelets in 100 μL of MTB). Blood sampling at the tail was collected at 1 min (baseline), 6 h, 12 h, 24 h and 48 h, and total platelets were labeled with PE-anti-CD41 antibody. The percentage of calcein-labeled platelets remaining in circulation was assessed by flow cytometry.

### Colony assay

Colony forming unit (CFU) assays were performed by culturing freshly sorted BM lin^-^c-kit^+^Sca-1^+^ cells on Methocult GF M3434 (StemCell Technologies). Methocult GF M3434 media (1 mL) containing 1000 lin^-^c-kit^+^Sca-1^+^ cells was plated and cultured for 7-10 days for CFU-erythroid (E), CFU-granulocyte/macrophage (GM), CFU-granulocyte (G), CFU-macrophage (M) and CFU-multipotential progenitor cell-granulocyte/erythrocyte/macrophage/megakaryocyte (GEMM) scoring. 2000 lin^-^c-kit^+^Sca-1^+^ cells were mixed with 1 mL of semisolid medium (Methocult-c, 04974, StemCell Technologies) supplemented with 10 ng/mL IL-3, 20 ng/mL IL-6 and 50 ng/mL TPO, and cultured for 6 days for CFU- megakaryocyte (MK) scoring.

### Real-time reverse transcription-polymerase chain reaction (real-time RT-PCR)

A total of 1 × 10^4^ sorted cells were lysed in lysis buffer (TaKaRa, 3735A) and subjected to real-time RT-PCR according to the instruments.

1 × 10^6^ sorted CD19^+^ B cells from either the *Aurka^f/f^* or *Aurka^f/f^;Cd19^Cre/+^* mice were exposed to either 10 mM C188-9 and/or 100 ng/mL IL-6. After 24 h, cells were harvested, RNA was extracted and subjected to real-time RT-PCR.

Hepatocytes were isolated from *Aurka^f/f^* mice treated with either control diluent or 1 µg IL-6. RNA was extracted and subjected to real-time RT-PCR. The primer sets for PCR are shown in Table 1.

### Immunoblotting

Immunoblotting was performed as previously described 30. Anti-Aurora-A (35C1, ab13824) antibody was purchased from Abcam (MA, USA). Anti-p-STAT1 (Tyr701)(D4A7, #7649), anti-STAT1 (D1K9Y, #14994), anti-p-STAT3 (Tyr705) (D3A7, #9145) and anti-STAT3 (D3Z2G, #12640) were purchased from Cell Signaling Technology (MA, USA). Anti-β-actin (66009-1-Ig) antibodies were purchased from Proteintech.

### Histologic analysis

Tissues were fixed in 4% formaldehyde and embedded in paraffin. Sections with a thickness of 4 µm were stained with hematoxylin-eosin (Beyotime Biotechnology, Nantong, Jiangsu, China) as described previously 31, 32.

### Immunofluorescence staining and quantification

Multiplex staining and multispectral imaging were performed to identify the cell subsets expressing CD41 (24552-1-AP, Proteintech), Mpl (bs-10362R, Bioss, dilution 1/50), IL-6 (bs-0782R, Bioss, dilution 1/50), TPO (Invitrogen, dilution 1/50), CD138 (10593-1-AP, Proteintech, dilution 1/50) or CD19 (D4V4B, #90176, Cell Signaling Technology, dilution 1/100) in the BM sections using a PANO 4-plex IHC kit (Yuanxi, Shanghai, China). Different primary antibodies were sequentially applied, followed by horseradish peroxidase-conjugated secondary antibody incubation and tyramide signal amplification. The slides were microwave heat-treated after each TSA operation. Nuclei were stained with 4', 6-diamidino-2-phenylindole (DAPI, Sigma) after all the mouse antigens had been labeled.

Images of BM sections were acquired using a Zeiss Axio Imager 2 microscope (×20 magnification) and analyzed using TissueFAXs and StrataQuest tissue analysis software (TissueGnostics, Beijing, China) as described previously 33. Briefly, identical exposure times and threshold settings were used for each channel on all sections of similar experiments. The algorithm detected nuclei on the basis of the signal from the DAPI channel, then expanded and built a mask over the cytoplasm 33. On the generated mask, the algorithm searched for the localization of 520, 570 and 650 signals. Results were plotted onto scattergrams, and events were manually verified for all quadrants. The number of CD41^+^Mpl^+^ megakaryocytes per area (mm^2^), and the number of CD19^+^ B cells or CD138^+^ plasma cells within the indicated distance with respect to megakaryocytes were calculated.

### Enzyme-linked immunosorbent assay (ELISA)

The concentrations of the indicated cytokines in the plasma were examined using ELISA kits (R&D) according to the manufacturer's instructions. The concentration of TPO in the plasma and in spleen or liver tissues was analyzed using an ELISA kit (mmBio, China) according to the manufacturer's instructions. Briefly, frozen spleen or liver tissues (20 - 30 mg) were weighed and homogenized in 50 mL PBS (pH 7.4). Homogenates were sonicated for 20 s and then centrifuged at 13200 rpm for 10 min at 4°C to remove cellular debris. After centrifugation, the supernatant was collected and subjected to ELISA. The concentration of TPO in tissues was calculated following the formula: (concentration × sample volume × diluent factor)/weight used for ELISA.

### ChIP assay

ChIP analysis was performed using a commercially available kit (Beyotime Biotechnology). DNA-bound proteins were crosslinked using formaldehyde at a final concentration of 1% for 20 min at 37°C. Protein-DNA complexes were immunoprecipitated using primary antibodies against STAT3 (#9145, Cell Signaling Technology). The binding sites of STAT3 on the TPO promoter were predicted using LASAGNA-Search 2.0: Searching for transcription factor binding sites (TFBSs) (https://biogrid-lasagna.engr.uconn.edu/lasagna_search). STAT3 and TPO promoter complexes were measured by PCR. The primer sets used for the amplification of the TPO promoter region between -1030 to -832 bp were as follows: forward 5'- GGGAAAACGATCCAACCAC-3', reverse 5'-GGAGGAGAGCAGGAGAAAGAA-3'; -3778 to -3637 bp without predicted STAT3 binding sites as a negative control, forward 5'-CTCTGGGGCTACTCTTGGTG-3', reverse 5'-ACCCTGATGTGCCTTGTTTC-3'. The samples were separated by electrophoresis in a 2% agarose gel and visualized by ethidium bromide staining.

### Correlation analysis using the GEPIA web tool

The online database Gene Expression Profiling Interactive Analysis (GEPIA2, http://gepia2.cancer-pku.cn/#index) was used to analyze a pairwise gene correlation analysis for any given set of expression data from the Genotype-Tissue Expression (GTEx) using Pearson correlation statistics.

### Statistical analysis

Statistical analysis was performed to assess the difference using an unpaired Student's t-test, two-way ANOVA followed by multiple comparisons by Prism statistical analysis software (GraphPad Software, San Diego, CA). Data are presented as the mean ± SD. Significance is indicated as follows: *P < 0.05 or n.s. for not significant.

## Results

### *Aurka* deficiency impaired B cell development

**Figure 1:**
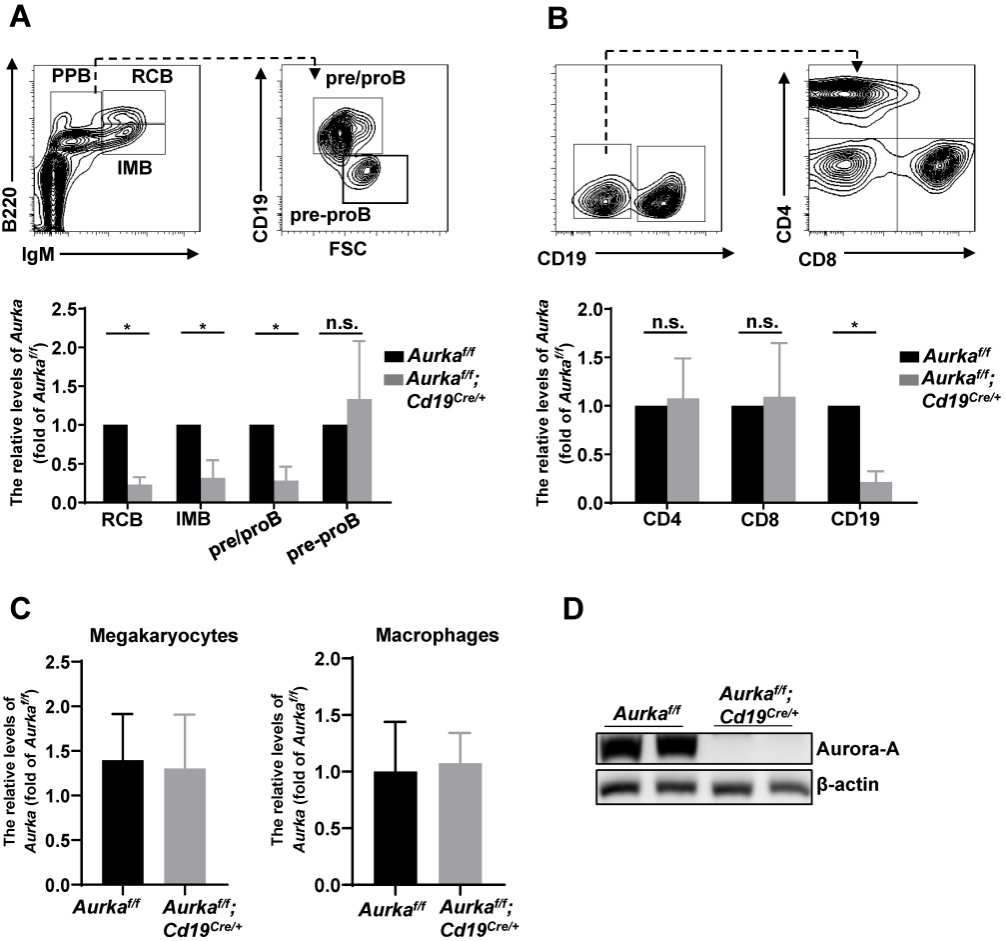
***Aurka* was deleted in CD19^+^ B cells. (A)** The mRNA levels of *Aurka* were examined in sorted RCB, IMB, pre/pro B and pre-pro B cells. *, *P* < 0.05, n.s., not significant.** (B)** The mRNA levels of *Aurka* were examined in sorted CD4^+^ T, CD8^+^ T and CD19^+^ B cells. *, *P* < 0.05, n.s., not significant.** (C)** The mRNA levels of *Aurka* were examined in sorted megakaryocytes and macrophages. (**A-C**) Data shown is mean ± SD of one of three independent experiments (n = 3 mice/group) with similar results. Significance was calculated using an unpaired Student's t-test. *, *P* < 0.05.** (D)** Aurora-A expression was examined in sorted CD19^+^ B cells from either *Aurka^f/f^* mice (n = 2) or *Aurka^f/f^;Cd19^Cre/+^* mice (n = 2) by Western blotting. The data shown is representative of one of two independent experiments.

To study the role of Aurora-A in B cells, we deleted the *Aurka* gene specifically in CD19-expressing B cells by crossing *Cd19^Cre/+^* mice with *Aurka^f/f^* mice. The *Aurka^f/f^;Cd19^Cre/+^* mice were born at the Mendelian ratio and phenotypically grossly normal (data not shown). We examined the *Aurka* mRNA levels by real-time RT-PCR and found that the levels of *Aurka* were obviously decreased in recirculating B cells (RCB), immature B cells (IMB) and pre/pro B cells (PPB) sorted from the *Aurka^f/f^;Cd19^Cre/+^* mice compared with those of the *Aurka^f/f^* mice (Figure 1A). However, the levels of *Aurka* did not change in pre-pro B cells without CD19 expression isolated from the BM of the *Aurka^f/f^;Cd19^Cre/+^* mice compared with the *Aurka^f/f^* mice (Figure 1A). As shown in Figure 1B, the levels of *Aurka* mRNA were dramatically reduced in the CD19-expressing B cells but not in the CD4- or CD8-expressing T cells. Additionally, the *Aurka* mRNA levels were almost identical in myeloid cells such as megakaryocytes and macrophages sorted from the *Aurka^f/f^;Cd19^Cre/+^* mice compared with those sorted from the *Aurka^f/f^* mice (Figure 1C). Western blot analysis further confirmed the decreased Aurora-A protein in the CD19-expressing B cells (Figure 1D).

**Figure 2:**
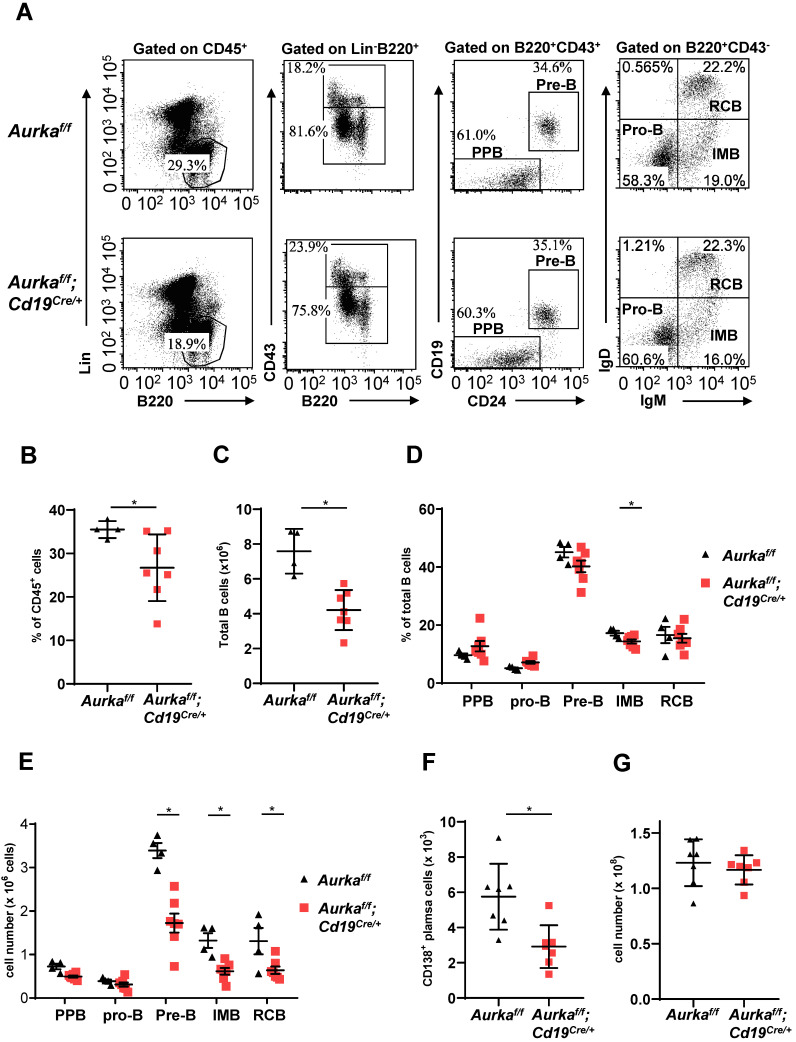
** B cell development was impaired in BM. (A)** The distribution of various B-cell populations in the BM of mice with different genotypes was assessed by flow cytometry. The data shown are representative of one of three independent experiments. **(B)** The dot graph shows the percentage of Lin^-^B220^+^ B cells gated on CD45^+^ cells in the BM. *, *P* < 0.05.** (C)** The dot graph shows the total number of Lin^-^B220^+^ B cells in the BM. *, *P* < 0.05. **(D)** The dot graph shows the percentage of various B-cell populations in the BM of mice with different genotypes. *, *P* < 0.05. **(E)** Dot graph shows the total number of various B-cell populations in the BM of mice with different genotypes. The total number of indicated cells was calculated following the following formula: the number of indicated B cells/the number of gated single cells × the total nucleated cells recovered from BM. *, *P* < 0.05.** (F)** CD19^+^ B cells (1 × 10^5^) in the BM of mice with different genotypes were analyzed. The dot graph shows the number of CD138^+^ plasma cells in these CD19^+^ B cells. *, *P* < 0.05.** (G)** The dot graph shows the total number of nucleated cells in the BM of mice with different genotypes. *, *P* < 0.05. **(B-G)** Data shown are mean ± SD of one of three independent experiments (n = 4 - 7 mice/group) with similar results. Significance was calculated using an unpaired Student's t-test.

B-cell development was impaired in the BM of the *Aurka^f/f^;Cd19^Cre/+^* mice. Compared with that of the *Aurka^f/f^* mice, the percentage of B220^+^ cells in gated CD45^+^ cells was obviously decreased (Figures 2A and 2B). The total number of B cells in BM was dramatically reduced in the *Aurka^f/f^;Cd19^Cre/+^* mice (Figure 2C). In addition, the percentage of IMB and the numbers of pre-B, IMB, RCB and CD138^+^ plasma cells were also significantly decreased in the BM of the *Aurka^f/f^;Cd19^Cre/+^* mice (Figures 2D-2F). However, the nucleated cell number in BM from the *Aurka^f/f^;Cd19^Cre/+^* mice was almost identical to that in BM from the *Aurka^f/f^* mice (Figure 2G).

**Figure 3:**
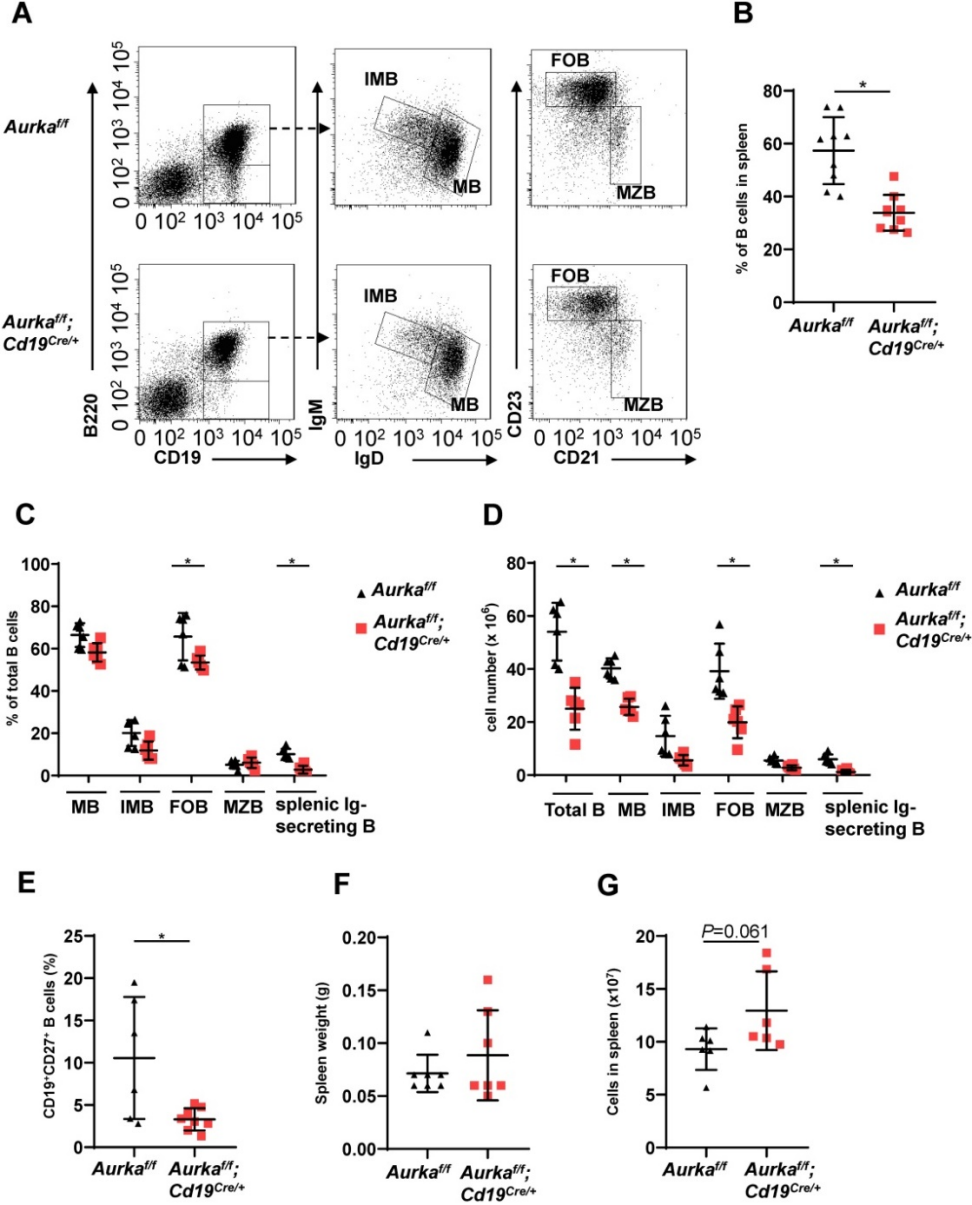
** B cell development was impaired in the spleen. (A)** Approximately 1 × 10^5^ nucleated cells in the spleens of mice with different genotypes were analyzed. The distribution of various B-cell populations in the spleen of mice with different genotypes was assessed by flow cytometry. The data shown is representative of one of two independent experiments. **(B)** The dot graph shows the percentage of CD19^+^ B cells gated on CD45^+^ cells in the spleens removed from the *Aurka^f/f^* mice or the *Aurka^f/f^;Cd19^Cre/+^* mice. Data shown are mean ± SD of one of two independent experiments (n = 9 mice/group) with similar results. *P*-value was calculated using an unpaired Student's t-test. *, *P* < 0.05.** (C)** The dot graph shows the percentage of various B-cell populations in the total B cells of the spleens removed from the *Aurka^f/f^* mice or the *Aurka^f/f^;Cd19^Cre/+^* mice. *, *P* < 0.05. **(D)** The dot graph shows the total number of indicated B cells in spleens. The total number of indicated cells was calculated with the following formula: the number of indicated B cells/the number of gated single cells × the total nucleated cells recovered from the spleen. *, *P* < 0.05.** (E)** CD19^+^ B cells (1 × 10^5^) in the spleens removed from the *Aurka^f/f^* mice or the *Aurka^f/f^;Cd19^Cre/+^* mice were analyzed. The dot graph shows the total number of CD27^+^ memory B cells in these CD19^+^ B cells. *, *P* < 0.05. **(C-E)** Data shown are mean ± SD of one of two independent experiments (n = 6 mice/group) with similar results. Significance was calculated using an unpaired Student's t-test. **(F)** The dot graph shows the spleen weight of either the *Aurka^f/f^* mice or the *Aurka^f/f^;Cd19^Cre/+^* mice. The data shown are representative of one of two independent experiments (n = 7 mice/group) with similar results. Significance was calculated using an unpaired Student's t-test. **(G)** The dot graph shows the total number of nucleated cells in the spleens of either the *Aurka^f/f^* mice or the *Aurka^f/f^;Cd19^Cre/+^* mice. The data shown are representative of one of two independent experiments (n = 6 mice/group) with similar results. Significance was calculated using an unpaired Student's t-test.

We next investigated the development of B cells in spleens and found that the percentage of B cells and the total number of B cells in spleens were potently decreased (Figures 3A-3C). As expected, the percentages of mature B (MB), IMB, follicular B (FOB) and splenic Ig-secreting B cells were lower in the *Aurka^f/f^;Cd19^Cre/+^* mice than in the *Aurka^f/f^* mice (Figure 3C). Additionally, the numbers of MB, IMB, FOB, marginal zone B (MZB) and splenic Ig-secreting B cells were decreased in the *Aurka^f/f^;Cd19^Cre/+^* mice compared with the *Aurka^f/f^* mice (Figure 3D). The percentages of CD27^+^ memory B cells were obviously lower in spleens isolated from the *Aurka^f/f^;Cd19^Cre/+^* mice than in those isolated from the *Aurka^f/f^* mice (Figure 3E). Unexpectedly, the weights of the spleens as well as the total splenic nucleated cell number were slightly increased in the *Aurka^f/f^;Cd19^Cre/+^* mice compared with the *Aurka^f/f^* mice (Figures 3F and 3G).

### *Aurka* loss in CD19^+^ B cells led to an increase in platelet production

**Figure 4:**
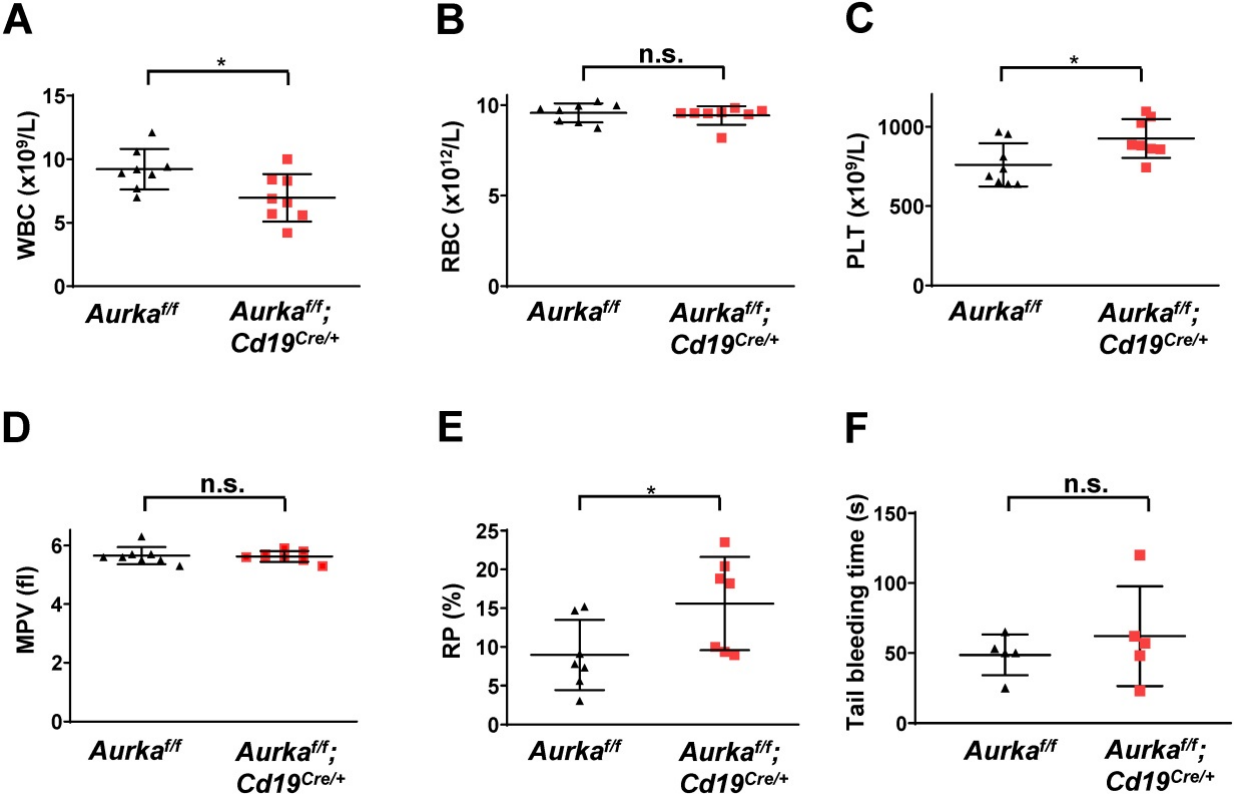
** The total number of platelets was increased in the *Aurka^f/f^;Cd19^Cre/+^* mice. (A)** The total number of WBCs in the peripheral blood of either the *Aurka^f/f^* mice or the *Aurka^f/f^;Cd19^Cre/+^* mice was examined by a Sysmex XP-100 hematologic analyzer. *, *P* < 0.05. **(B)** Dot graph shows the total number of RBCs in the peripheral blood of either the *Aurka^f/f^* mice or the *Aurka^f/f^;Cd19^Cre/+^* mice. n.s., not significant. **(C)** The dot graph shows the total number of platelets in the peripheral blood of either the *Aurka^f/f^* mice or the *Aurka^f/f^;Cd19^Cre/+^* mice. *, *P* < 0.05. **(D)** The dot graph shows the mean platelet volume. n.s., not significant. **(A-D)** The data shown are representative of one of two independent experiments (n = 8 mice/group) with similar results. Significance was calculated using an unpaired Student's t-test.** (E)** The dot graph shows the percentage of RPs in approximately 3 × 10^4^ platelets isolated from either the *Aurka^f/f^* mice or the *Aurka^f/f^;Cd19^Cre/+^* mice. The data shown are representative of one of three independent experiments (n = 5 - 8 mice/group) with similar results. Significance was calculated using an unpaired Student's t-test. *, *P* < 0.05. **(F)** Tail bleeding time analysis was used to analyze platelet function *in vivo*. The data shown are representative of one of two independent experiments (n = 5 mice/group) with similar results. Significance was calculated using an unpaired Student's t-test. n.s., not significant.

To further confirm whether deletion of Aurora-A in CD19-expressing cells could induce a decrease in the number of white blood cells (WBCs), we performed blood cell counts and found that the total number of WBCs was obviously decreased (Figure 4A). Deletion of *Aurka* did not influence the number of red blood cells (RBCs, Figure 4B). Unexpectedly, the total platelet count was elevated in the *Aurka^f/f^;Cd19^Cre/+^* mice compared with of the *Aurka^f/f^* mice or the *Aurka^+/+^;Cd19^Cre/+^* mice (Figures 4C and S1). Additionally, the percentage of RP was also increased in the *Aurka^f/f^;Cd19^Cre/+^* mice compared with of the *Aurka^f/f^* mice, although the mean platelet volume was identical in the *Aurka^f/f^;Cd19^Cre/+^* mice and the *Aurka^f/f^* mice (Figures 4D and 4E). We also investigated whether *Aurka* loss in CD19^+^ B cells influenced platelet function *in vivo*. We performed a tail bleeding assay and found that the *Aurka^f/f^;Cd19^Cre/+^* mice demonstrated slightly increased tail bleeding times compared with the *Aurka^f/f^* mice (Figure 4F).

### Deletion of *Aurka* in CD19^+^ B cells promoted thrombopoiesis

**Figure 5:**
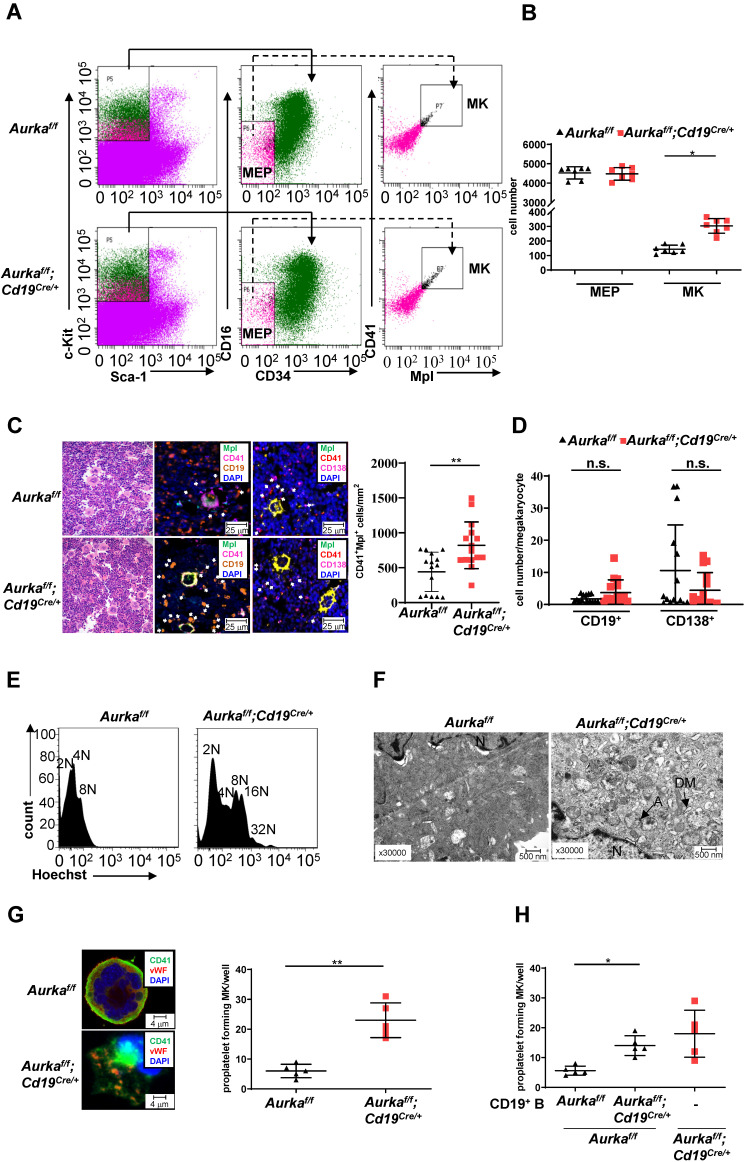
***Aurka* loss increased the number of CD41^+^Mpl^+^ megakaryocytes and megakaryocyte differentiation. (A)** Approximately 3 × 10^4^ Lin^-^c-Kit^+^Sca-1^-^ cells were analyzed. The distribution of MEPs and megakaryocytes in the BM of mice with different genotypes was assessed by flow cytometry. The data shown are representative of one of two independent experiments.** (B)** The dot graph shows the number of MEPs and megakaryocytes in BMs removed from the *Aurka^f/f^* mice or the *Aurka^f/f^;Cd19^Cre/+^* mice. The data shown are representative of one of two independent experiments (n = 7 mice/group) with similar results. Significance was calculated using an unpaired Student's t-test. *, *P* < 0.05. **(C, left)** H&E and Immunofluorescence staining for CD41^+^Mpl^+^ megakaryocytes, CD19^+^ B cells, or CD138^+^ plasma cells in the BM sections from the *Aurka^f/f^* or *Aurka^f/f^;Cd19^Cre/+^* mice. **(C, right)** The dot graph shows the number of CD41^+^Mpl^+^ megakaryocytes in the BM sections from the *Aurka^f/f^* mice or the *Aurka^f/f^;Cd19^Cre/+^* mice. The data shown is representative of one of two independent experiments (n = 3 mice/group, 5 slides/mouse) with similar results. *P* values were obtained using a 2-sided, unpaired Student's t-test. **, *P* < 0.01. **(D)** Dot graph shows the number of CD19^+^ B cells or CD138^+^ plasma cells within the 25 mm distance with respect to megakaryocytes in the BM sections from the *Aurka^f/f^* mice or the *Aurka^f/f^;Cd19^Cre/+^* mice. The data shown is representative of one of two independent experiments (n = 3 mice/group, 5 slides/mouse) with similar results. Significance was calculated using an unpaired Student's t-test. n.s., not significant. **(E)** The ploidy of megakaryocytes was studied after staining with anti-CD41, anti-Mpl antibodies and Hoechst labeling. The histograms show one of three independent experiments (n = 5 mice/group) with similar results. **(F)** The ultrastructure of megakaryocytes was visualized by TEM. DM, demarcation membrane; N, nucleus; A, α-granules. The data shows one of three independent experiments (n = 3 mice/group) with similar results.** (G)** Lin^-^c-Kit^+^ cells from the BM of either the *Aurka^f/f^* mice or the *Aurka^f/f^;Cd19^Cre/+^* mice were cultured in the presence of 200 ng/mL mTPO for 4 days. **(Left)** Representative staining of megakaryocytes with anti-CD41 (green) and anti-vWF (red) antibodies was shown. **(Right)** Proplatelet forming megakaryocytes were counted. The data shows one of two independent experiments (n = 3 - 5 mice/group) with similar results. Significance was calculated using an unpaired Student's t-test. **, *P* < 0.01. **(H)** Lin^-^c-Kit^+^ cells from the BM of the *Aurka^f/f^* mice were cultured with CD19^+^ B cells sorted from either the *Aurka^f/f^* mice or the *Aurka^f/f^;Cd19^Cre/+^* mice in the presence of 200 ng/mL mTPO together with murine IL-17 for 4 days. Proplatelet-forming megakaryocytes were counted. The dot graph shows one of two independent experiments with similar results. Significance was calculated using an unpaired Student's t-test. * *P* < 0.05.

Deletion of *Aurka* in hematopoietic cells or inhibition of Aurora-A activity by its inhibitor promoted the differentiation of megakaryocytes 1. To determine whether the observed elevated platelets in the *Aurka^f/f^;Cd19^Cre/+^* mice were linked to an increase in megakaryocyte progenitor cells as well as differentiated megakaryocytes, we first examined the percentages of Lin^-^c-Kit^+^Sca1^-^CD34^-^CD16^-^ cells that enrich bipotential precursors (MEPs) 34. As shown in Figures 5A and 5B, the number of MEPs was almost identical in the *Aurka^f/f^;Cd19^Cre/+^* mice and the *Aurka^f/f^* mice. Interestingly, the number of CD41^+^Mpl^+^ megakaryocytes was significantly increased in the *Aurka^f/f^;Cd19^Cre/+^* mice compared with the *Aurka^f/f^* mice. Multiplex immunofluorescence staining also revealed that the number of CD41^+^Mpl^+^ megakaryocytes was obviously elevated in the *Aurka^f/f^;Cd19^Cre/+^* mice compared with the *Aurka^f/f^* mice (Figure 5C). A previous study showed that megakaryocytes interact with plasma cells and are an important component of the niche for long-lived plasma cells in the BM 35. However, whether CD19^+^ B cells or plasma cells are critical for megakaryocytes is unclear. We therefore examined the localization of CD19^+^ B cells with respect to megakaryocytes in BM and found that the cell number of CD19^+^ B cells surrounding CD41^+^Mpl^+^ megakaryocytes (within 25 mm) was higher in the BM of the *Aurka^f/f^;Cd19^Cre/+^* mice than in that of the *Aurka^f/f^* mice (Figure 5D). However, the number of CD138^+^ plasma cells surrounding CD41^+^Mpl^+^ megakaryocytes (within 25 mm) was lower in the BM of the *Aurka^f/f^;Cd19^Cre/+^* mice than in that of the *Aurka^f/f^* mice (Figure 5D).To further confirm the megakaryocyte differentiation observed in the *Aurka^f/f^;Cd19^Cre/+^* mice, we examined the ploidy and ultrastructure of megakaryocytes. An increase in megakaryocyte ploidy was observed in the *Aurka^f/f^;Cd19^Cre/+^* mice compared with the *Aurka^f/f^* mice (Figure 5E). Moreover, well-developed demarcation membranes (DMs) were observed in cultured megakaryocytes from the *Aurka^f/f^;Cd19^Cre/+^* mice compared with those from the *Aurka^f/f^* mice (Figure 5F).

The above results indicated that *Aurka* loss in CD19^+^ B cells could influence the late stage of megakaryocyte differentiation. Therefore, we assessed proplatelet formation. As shown in Figure 5G, an increase in the number of proplatelet formations was observed in megakaryocytes from the *Aurka^f/f^;Cd19^Cre/+^* mice. To further investigate whether *Aurka* deficiency in CD19^+^ B cells promoted thrombopoiesis, we cocultured Lin^-^c-Kit^+^ hematopoietic progenitors from the *Aurka^f/f^* mice with CD19^+^ B cells sorted from either the* Aurka^f/f^* or *Aurka^f/f^;Cd19^Cre/+^* mice. We found that the number of proplatelet-forming megakaryocytes was obviously increased when Lin^-^c-Kit^+^ hematopoietic progenitors from the *Aurka^f/f^* mice were cocultured with CD19^+^ B cells from the *Aurka^f/f^;Cd19^Cre/+^* mice (Figure 5H).

To further test whether the proliferative status of hematopoietic stem cells (HSCs) was impaired and HSCs tended toward a megakaryocyte bias in the BMs of the *Aurka^f/f^;Cd19^Cre/+^* mice, we performed a colony assay. Compared to that of the *Aurka^f/f^* mice, the proliferative status of Lin^-^c-Kit^+^Sca-1^+^ cells, which enrich HSCs, was not affected in the *Aurka^f/f^;Cd19^Cre/+^* mice (Figure S2A). Compared with Lin^-^c-Kit^+^Sca-1^+^ cells from the *Aurka^f/f^* mice, Lin^-^c-Kit^+^Sca-1^+^ cells from the *Aurka^f/f^;Cd19^Cre/+^* mice did not show an obvious bias toward megakaryocyte colonies (Figure S2B). To clarify whether the higher numbers of megakaryocytes in the BM of the *Aurka^f/f^;Cd19^Cre/+^* mice were associated with an increase in megakaryocytopoiesis in the spleens, we examined the population of megakaryocytes in spleens. The number of CD41^+^Mpl^+^ cells was increased in spleens (Figure S2C), although the spleen weights were slightly increased (Figure 3F).

### Deletion of *Aurka* in CD19^+^ B cells induced TPO production

**Figure 6:**
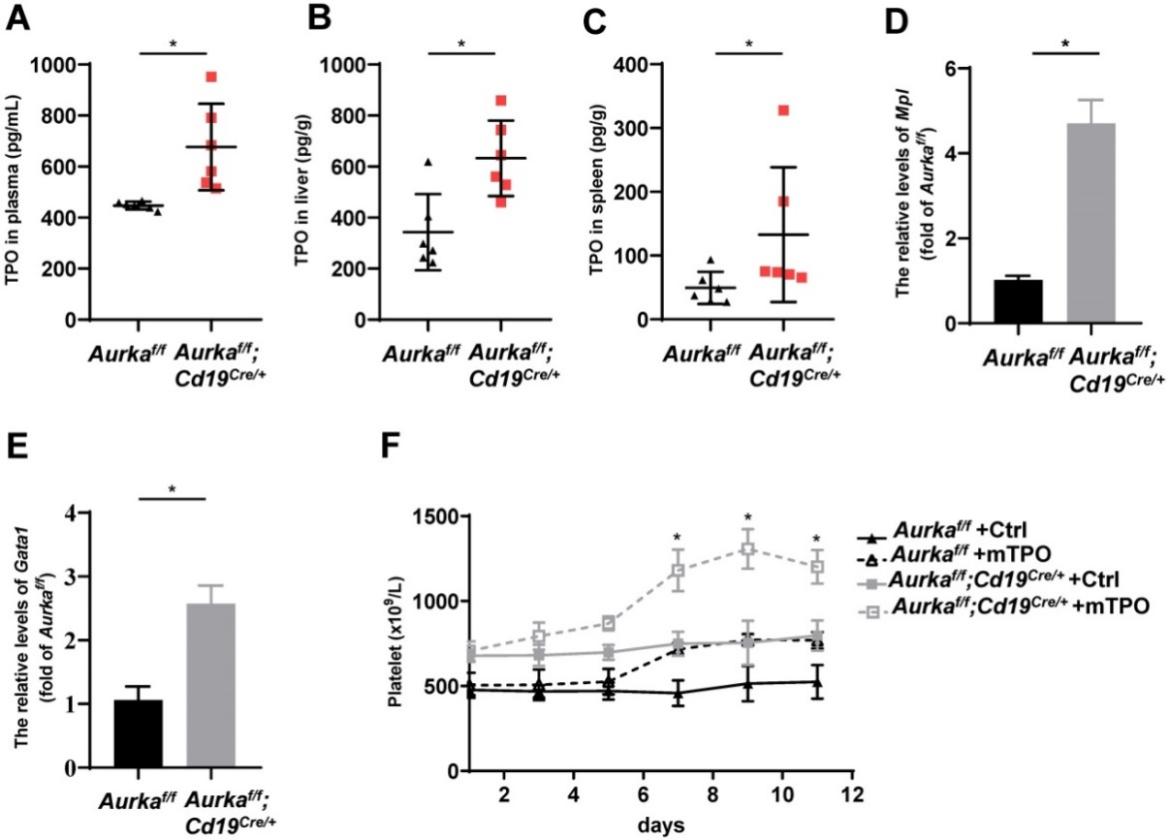
***Aurka* deficiency in CD19^+^ B cells induced TPO production. (A)** The concentrations of TPO in the plasma were examined by ELISA. * *P* < 0.05.** (B)** Frozen livers were weighed, homogenized, and followed by sonication. After centrifugation, the supernatant was collected and subjected to ELISA to examine the concentrations of TPO. The concentrations of TPO in the liver were calculated following the formula: (concentration × sample volume × diluent factor)/weight. * *P* < 0.05.** (C)** Frozen spleen were weighed, homogenized, and followed by sonication. After centrifugation, the supernatant was collected and subjected to ELISA. The concentrations of TPO in the spleen were examined by ELISA, and calculated following the formula: (concentration × sample volume × diluent factor)/weight. * *P* < 0.05. **(A-C)** The dot graphs show one of two independent experiments (n = 6 mice/group) with similar results. *P*-value was calculated using an unpaired Student's t-test. **(D)** Real-time RT-PCR to quantify the *Mpl* mRNA levels in sorted CD41^+^Mpl^+^ megakaryocytes from the *Aurka^f/f^* or *Aurka^f/f^;Cd19^Cre/+^* mice. *P < 0.05.** (E)** Real-time RT-PCR to quantify *Gata1* mRNA levels in the sorted CD41^+^Mpl^+^ megakaryocytes from the *Aurka^f/f^* or *Aurka^f/f^;Cd19^Cre/+^* mice. * *P* < 0.05.** (D-E)** The graphs show one of two independent experiments (n = 3 mice/group) with similar results. Significance was calculated using an unpaired Student's t-test.** (F)** The graph shows the total number of platelets in the peripheral blood of the indicated mice treated with either control diluent or mTPO. The data shows one of two independent experiments (n = 5 mice/group) with similar results. Significance was calculated using two way ANOVA followed by Tukey's multiple comparisons test. *, *P* < 0.05.

TPO has been identified as the most important regulator of megakaryocyte maturation and platelet production signaling via its receptor, c-Mpl 36. We analyzed the levels of TPO in plasma and tissues. We found that the concentration of TPO was increased in the plasma of the *Aurka^f/f^;Cd19^Cre/+^* mice compared with the *Aurka^f/f^* mice (Figure 6A). Unexpectedly, the concentration of TPO was increased in the livers and spleens of the *Aurka^f/f^;Cd19^Cre/+^* mice compared with those of the *Aurka^f/f^* mice (Figures 6B and 6C). We next examined Mpl expression in sorted CD41^+^Mpl^+^ megakaryocytes and found that the mRNA levels of *Mpl* were markedly upregulated in the CD41^+^Mpl^+^ megakaryocytes sorted from the* Aurka^f/f^;Cd19^Cre/+^* mice (Figure 6D). In addition, the mRNA levels of *Gata1*, a transcription factor that is essential for proper growth and differentiation of megakaryocytes, were significantly increased in the *Aurka^f/f^;Cd19^Cre/+^* mice compared with the *Aurka^f/f^* mice (Figure 6E). To further investigate whether the elevated platelets were the consequence of megakaryocyte maturation mediated by an increase in TPO production, we treated the *Aurka^f/f^;Cd19^Cre/+^* mice and the *Aurka^f/f^* mice with either control diluent or mTPO. We found that upon mTPO treatment, the number of platelets was increased in the *Aurka^f/f^;Cd19^Cre/+^* mice compared with the *Aurka^f/f^* mice (Figure 6F).

### IL-6 was critical for TPO production

**Figure 7:**
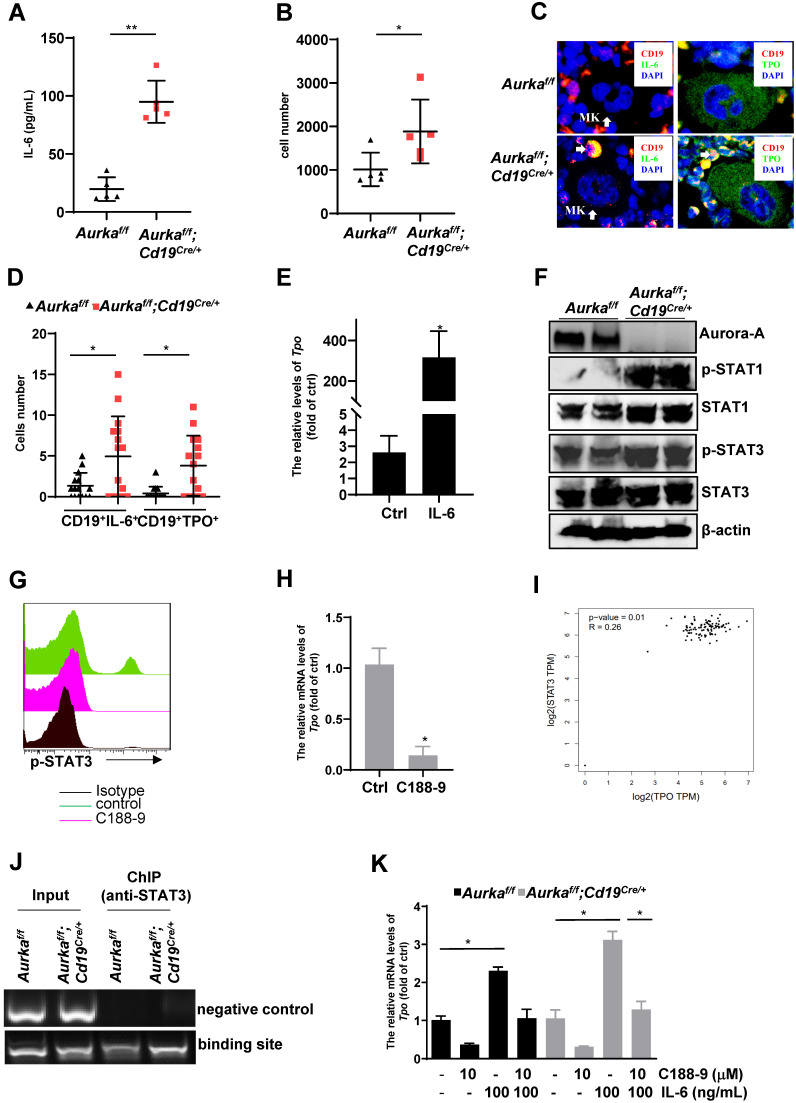
** IL-6-activated STAT3 was required for *Tpo* transcription. (A)** The concentration of IL-6 in the plasma was examined by ELISA. The data shows one of two independent experiments (n = 5 - 6 mice/group) with similar results. **P < 0.01.** (B)** CD19^+^ B cells (3 × 10^4^) in the spleens of the *Aurka^f/f^* mice or the *Aurka^f/f^;Cd19^Cre/+^* mice were analyzed. The dot graph shows the total number of IL-6-positive cells in these CD19^+^ B cells. The data shows one of two independent experiments (n = 5 mice/group) with similar results. *, *P* < 0.05. **(C, left)** Immunofluorescence for CD19 (red), IL-6 (green) and DAPI (blue) in the BM sections from the *Aurka^f/f^* or *Aurka^f/f^;Cd19^Cre/+^* mice. Mk, megakaryocyte. **(C, right)** Immunofluorescence for CD19 (red), TPO (green) and DAPI (blue) in the BM sections from the *Aurka^f/f^* or *Aurka^f/f^;Cd19^Cre/+^* mice. The data shows one of two independent experiments with similar results.** (D)** Dot graph of **Figure 7C** shows the number of CD19^+^IL-6^+^ B cells, or CD19^+^TPO^+^ B cells close to megakaryocytes in the BM sections from the *Aurka^f/f^* mice (n = 3 mice/group, 5 slides/mouse) or the *Aurka^f/f^;Cd19^Cre/+^* mice (n = 3 mice/group, 5 slides/mouse). *, *P* < 0.05. **(E)** *Aurka^f/f^* mice were treated with either control diluent or 1 µg IL-6. After which, hepatocytes were isolated and subjected to real-time RT-PCR to quantify the *Tpo* mRNA levels. The data shows one of two independent experiments (n = 5 mice/group) with similar results. *, *P* < 0.05. **(A-E)** *P*-values were obtained using a 2-sided, unpaired Student's t-test. **(F)** The expression of the indicated protein was examined in spleens from either the *Aurka^f/f^* or *Aurka^f/f^;Cd19^Cre/+^* mice by Western blotting. **(G)** CD19^+^ B cells were sorted from either the *Aurka^f/f^* or *Aurka^f/f^;Cd19^Cre/+^* mice. 5 × 10^5^/mL sorted CD19^+^ B cells were exposed to 10 mM C188-9. After 24 h, cells were harvested, fixed and subjected to FACS to examine the levels of p-STAT3. **(F-G)** The data shows one of two independent experiments.** (H)** CD19^+^ B cells were sorted from either the *Aurka^f/f^* or *Aurka^f/f^;Cd19^Cre/+^* mice. 1 × 10^6^/mL sorted CD19^+^ B cells were exposed to 10 mM C188-9. After 24 h, cells were harvested, RNA was extracted and subjected to real-time RT-PCR to quantify the *Tpo* mRNA levels. Data shown is mean ± SD of one of two independent experiments (n = 3 mice/group) with similar results. Significance was calculated using an unpaired Student's t-test. *, *P* < 0.05. **(I)** The correlation of STAT3 and TPO was assessed by GEPIA.** (J)** CD19^+^ B cells were sorted from either the *Aurka^f/f^* or *Aurka^f/f^;Cd19^Cre/+^* mice. After being crosslinked with formaldehyde, protein-DNA complexes were immunoprecipitated using an anti-STAT3 antibody. After which, DNA was extracted and subjected to PCR to measure the STAT3 binding sites on the TPO promoter. The PCR products were separated in a 2% agarose gel and visualized by ethidium bromide staining. bp, base pair. Data shows one of two independent experiments with similar results. **(K)** CD19^+^ B cells were sorted from either the *Aurka^f/f^* or *Aurka^f/f^;Cd19^Cre/+^* mice. 1 × 10^6^/mL sorted CD19^+^ B cells were exposed to either 10 mM C188-9 and/or 100 ng/mL IL-6. After 24 h, cells were harvested, RNA was extracted and subjected to real-time RT-PCR to quantify the *Tpo* mRNA levels. Data shown is mean ± SD of one of two independent experiments (n = 3 mice/group) with similar results. Significance was calculated using an unpaired Student's t-test. *P < 0.05.

IL-6 promotes thrombopoiesis by mediating TPO mRNA levels 10. To further investigate the mechanisms involved in regulating megakaryocyte differentiation in the *Aurka^f/f^;Cd19^Cre/+^* mice, we examined the concentration of IL-6, and we found that compared with that of the *Aurka^f/f^* mice, the concentration of IL-6 was increased in the plasma harvested from the *Aurka^f/f^;Cd19^Cre/+^* mice (Figure 7A). Given that a relatively lower number of B cells was observed in the *Aurka^f/f^;Cd19^Cre/+^* mice and that IL-6 promotes hepatocyte TPO production, we assumed that *Aurka* loss in CD19^+^ B cells could contribute to IL-6 expression, which promotes hepatocyte TPO expression. To clarify the tissue origin of these cytokines present in the plasma, we first analyzed the population of CD19^+^ B cells expressing IL-6 in spleens and PB and found that the number of CD19^+^IL-6^+^ B cells was increased in the spleens and PB of the *Aurka^f/f^;Cd19^Cre/+^* mice compared with the *Aurka^f/f^* mice (Figures 7B and S3A). Similarly, in the BM of the *Aurka^f/f^* mice, IL-6-secreting B cells were barely detected, whereas CD19^+^IL-6^+^ B cells were close to megakaryocytes in the BM of the *Aurka^f/f^;Cd19^Cre/+^* mice (Figure 7C, left panel and Figure 7D).

Unexpectedly, TPO-secreting B cells were found around megakaryocytes in the BM of the *Aurka^f/f^;Cd19^Cre/+^* mice compared with the *Aurka^f/f^* mice (Figure 7C, right panel and Figure 7D). We next treated the *Aurka^f/f^* mice with either control diluent or IL-6. As shown in Figure 7E, compared with those of the *Aurka^f/f^* mice treated with control diluent, the mRNA levels of *Tpo* were dramatically increased in the hepatocytes sorted from the *Aurka^f/f^* mice treated with IL-6. These observations suggested that *Aurka* deficiency in CD19^+^ B cells led to upregulation of IL-6, which was critical for hepatocyte TPO production. Additionally, *Aurka* loss could induce an increase in TPO expression in CD19^+^ B cells.

### STAT3 activation was critical for TPO transcription in *Aurka-*deficient B cells

STAT3 is the main downstream effector molecule of IL-6 37. STAT3 plays an important role in Ashwell-Morell receptor-induced hepatic TPO transcription 14. We therefore explored whether STAT3 was activated and required for TPO transcription. As we expected, the levels of the phosphorylated forms of STAT3 were upregulated in the spleen tissues of the *Aurka^f/f^;Cd19^Cre/+^* mice compared to those of the *Aurka^f/f^* mice (Figure 7F). In addition, consistent with our unpublished data, the total amount of STAT1 was upregulated in the spleen tissues of the *Aurka^f/f^;Cd19^Cre/+^* mice compared to that of the *Aurka^f/f^* mice (Figure 7F). To confirm the role of activated STAT3 in TPO expression, we treated CD19^+^ B cells sorted from the *Aurka^f/f^;Cd19^Cre/+^* mice with C188-9, a STAT3 inhibitor (Figure 7G). As shown in Figure 7H, the levels of *Tpo* mRNA were reduced in these cells. We also analyzed the correlation of STAT3 and TPO using the GEPIA web tool. Our data indicated that the expression of STAT3 was positively correlated with the expression of TPO (Figure 7I). To explore whether STAT3 directly mediated TPO transcription, we performed a ChIP assay and found that STAT3 bound to the TPO promoter (Figure 7J).

To further investigate whether STAT3-mediated TPO expression in *Aurka*-deficient B cells is an autocrine effect provoked by IL-6, we treated CD19^+^ B cells sorted from either the *Aurka^f/f^* mice or* Aurka^f/f^;Cd19^Cre/+^* mice with 100 ng/mL IL-6 and/or 10 mM C188-9. IL-6 (100 ng/mL) induced an increase in the mRNA levels of *Tpo* in sorted CD19^+^ B cells, which was attenuated in the presence of C188-9 (Figure 7K). These observations indicated that STAT3-mediated TPO transcription could be due to the autocrine effect provoked by IL-6.

### *Aurka* deficiency did not affect platelet clearance

**Figure 8:**
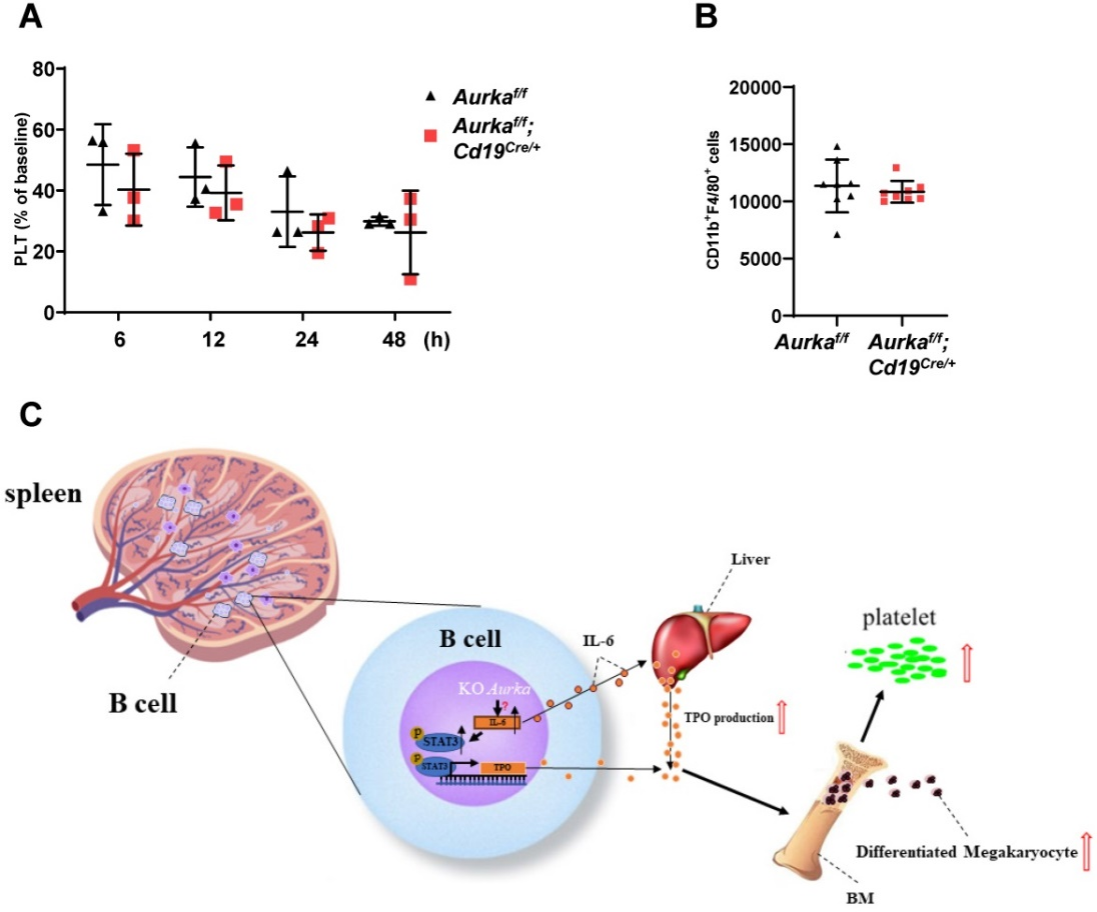
** Platelet clearance was not affected. (A)** Isolated platelets from either the *Aurka^f/f^* or* Aurka^f/f^;Cd19^Cre/+^* mice were incubated with calcein and transfused into the *Aurka^f/f^* mice. The percentage of calcein-positive platelets remaining in circulation was assessed at the indicated time points by FACS; Data shown is mean ± SD of one of two independent experiments (n = 3 - 5 mice/group) with similar results. Baseline was defined as the percentage of calcein-positive platelets within 1 min after platelet transfusion. **(B)** The population of macrophages in the CD45^+^ cells (1 × 10^5^) of the spleen was examined by FACS. The graph shows the mean ± SD of one of two independent experiments (n = 8 mice/group) with similar results. **(C)** Schematic representation of *Aurka* deficiency-mediated *Tpo* transcription and platelet production in the *Aurka^f/f^;Cd19^Cre/+^* mice. In CD19^+^ B cells, silencing *Aurka* led to an increase in IL-6 production, inducing STAT3 activation, which contributed to *Tpo* transcription and production; additionally, elevated IL-6 enhanced TPO production in the liver, which in turn promoted megakaryocyte differentiation, leading to an increase in platelet production.

To investigate whether platelet clearance was impaired in the *Aurka^f/f^;Cd19^Cre/+^* mice, we injected calcein-labeled platelets isolated from the *Aurka^f/f^* mice into the *Aurka^f/f^;Cd19^Cre/+^* mice. Platelet clearance was not impaired in either the *Aurka^f/f^;Cd19^Cre/+^* mice or the *Aurka^f/f^* mice (Figure 8A). Previous studies have indicated that splenic macrophages are involved in mediating platelet clearance 24. We therefore examined the population of macrophages and found that the number of splenic macrophages was identical in the *Aurka^f/f^;Cd19^Cre/+^* mice compared with the *Aurka^f/f^* mice (Figure 8B).

## Discussion

Aurora-A, one of the members of the Aurora family, plays an essential role in mitosis. Its overexpression has also been implicated in the pathogenesis of various hematologic neoplasms, including highly aggressive B-cell non-Hodgkin's lymphomas 38, 39. Suppression of Aurora-A resulted in defects in mitotic spindle assembly 40. In our study, we showed that *Aurka* deficiency in CD19*^+^* B cells significantly reduced the number of total B cells and subsets of B cells, including pre-B cells, IMB cells and FOB cells, in the BM or spleen (Figures 2-3). The number of memory B cells and plasma cells was also decreased in the *Aurka^f/f^;Cd19^Cre/+^* mice compared with the *Aurka^f/f^* mice (Figures 2F and 3E). Additionally, our unpublished data indicated that antibody maturation and class switching were abrogated in the *Aurka^f/f^;Cd19^Cre/+^* mice compared with the *Aurka^f/f^* mice. These observations indicated that loss of *Aurka* in CD19*^+^* B cells impaired the development and homeostasis of B cells, which could be the consequence of defects in mitotic spindle assembly.

A growing number of studies have shown that silencing Aurora-A in hematopoietic cells or inhibition of Aurora-A activity by its specific inhibitors induced the differentiation of megakaryocytes, in contrast to the survival defects of other hematopoietic lineages 1, 5, indicating that Aurora-A is required for adult hematopoiesis rather than the polyploidization and differentiation of megakaryocytes 1. In this study, an increase in the number of platelets and RP was also observed in the *Aurka^f/f^;Cd19^Cre/+^* mice, without influencing the mean volume of platelets or platelet function* in vivo*, compared with the *Aurka^f/f^* mice (Figures 4C-4F). Interestingly, the cellularity was almost identical in the *Aurka^f/f^;Cd19^Cre/+^* mice and the *Aurka^f/f^* mice (Figure 2G). However, the population of CD41^+^Mpl^+^ megakaryocytes, but not Lin^-^c-Kit^+^Sca1^-^CD34^-^CD16^-^ cells, which enrich BEMPs, was significantly elevated in the BM of the *Aurka^f/f^;Cd19^Cre/+^* mice compared with the *Aurka^f/f^* mice (Figures 5A-5C). Additionally, no impaired hematopoiesis or obvious bias toward megakaryocyte colonies was observed in the *Aurka^f/f^;Cd19^Cre/+^* mice (Figures S2A and S2B), suggesting that deletion of *Aurka* in CD19^+^ B cells contributed to elevated platelet numbers by indirectly influencing the late stage of megakaryocyte differentiation. Additionally, IL-6 plays an important role in mediating thrombopoiesis. The presence of STAT3 inhibitors not only blocked MPO pathway but also IL-6 pathway. We therefore, cannot rule out the possibility that elevated IL-6 contributed to megakaryocytopoiesis**.** Interestingly, the level of IL-6 was obviously higher in the plasma of *Aurka^f/f^;Cd19^Cre/+^* mice as compared with that of *Aurka^f/f^* mice (Figure 7A). Whereas, in the spleens, the number of IL-6 producing B cells was modestly increased in *Aurka^f/f^;Cd19^Cre/+^* mice as compared with *Aurka^f/f^* mice (Figures 7B and S3A), which implicated that the non-B cells expressing IL-6, such as macrophages could be involved in IL-6 production. To address the question, we examined the number of IL-6 producing macrophages and found that the population of IL-6 producing macrophages was higher in BMs and spleens of *Aurka^f/f^;Cd19^Cre/+^* mice as compared with *Aurka^f/f^* mice (Figures S3A and S3B). The mechanisms by which the number of macrophages expressing IL-6 was induced needed to be further investigated.

In this study, we found that deletion of *Aurka* in CD19^+^ B cells contributed to megakaryocytopoiesis in the spleen (Figure S2C). Moreover, ploidy assays (Figure 5E) and the ultrastructure of megakaryocytes assessed by transmission electron microscopy analysis (Figure 5F) revealed that loss of *Aurka* in CD19^+^ B cells promoted megakaryocyte differentiation. BEMPs are indeed characterized as Lin^-^c-Kit^+^Sca1^-^CD34^-^CD16^-^ cells 41, 42. A study demonstrated that *in vitro*, Lin^-^cKit^+^Sca1^-^IL7Rα^-^FcγRII/III^lo^CD150^+^ cells generated an increase in the proportion of megakaryocytes and erythroid cells in the response to TPO and EPO, respectively, although these cells proliferated less than their bipotential erythroid-megakaryocyte progenitors (BEMPs) counterparts 34, indicating that the cultured Lin^-^cKit^+^Sca1^-^IL7Rα^-^FcγRII/III^lo^CD150^+^ cells could be appropriate to described BEMPs among freshly isolated LSK cells.

A previous study demonstrated that megakaryocytes are an important component of the niche for long-lived plasma cells in the BM 35. Unexpectedly, the number of CD19^+^ B cells, but not plasma cells close to megakaryocytes, was higher in the BM of the *Aurka^f/f^;Cd19^Cre/+^* mice (Figure 5D), indicating that these cells could be involved in mediating megakaryocyte differentiation. Notably, in contrast to the *Aurka^f/f^* mice in which IL-6-secreting CD19^+^ B cells or TPO-secreting CD19^+^ B cells were barely detected around megakaryocytes, the number of IL-6-secreting CD19^+^ B cells or TPO-secreting CD19^+^ B cells relatively close to megakaryocytes was higher in the BM of the *Aurka^f/f^;Cd19^Cre/+^* mice (Figures 7C and 7D). In combination with the relatively higher levels of TPO and IL-6 in the plasma of the *Aurka^f/f^;Cd19^Cre/+^* mice (Figures 6A and 7A), these data indicated that TPO and IL-6 could be involved in mediating thrombopoiesis in the *Aurka^f/f^;Cd19^Cre/+^* mice(Figure 8C).

Consistent with previous studies, IL-6 induced TPO production 10 and directly increased the platelet count by mediating megakaryocyte maturation *in vivo* 43. Our results also demonstrated that IL-6 was responsible for the increased *Tpo* mRNA levels in hepatocytes (Figure 7E). Additionally, IL-6 induced upregulation of *Tpo* mRNA levels in CD19^+^ B cells, although the elevated levels were lower than those in hepatocytes, but this change was attenuated by the STAT3 inhibitor C188-9 (Figures 7H and 7K), suggesting that *Aurka* loss in CD19^+^ B cells promoted *Tpo* transcription via STAT3 activation, at least partially.

As a transcription factor, STAT3 plays a pivotal role in many cellular processes, including oncogenesis, tumor growth and stemness, by positively or negatively mediating numerous target genes. In HepG2 cells, suppression of the Jak2/STAT3 signaling pathway reduced the levels of *Tpo* mRNA mediated by AMR 10. In this study, we found that there were STAT3 binding sites on the TPO promoter (Figure 7J). STAT3 is a key mediator of the IL-6 signaling pathway. Together with the above data that higher levels of IL-6 were observed in *Aurka^f/f^;Cd19^Cre/+^* mice, STAT3-mediated TPO expression in *Aurka*-deficient B cells seemed to be an autocrine effect provoked by IL-6.

Nuclear Aurora-A functions as a transcription factor by interacting with heterogeneous nuclear ribonucleoprotein K (hnRNP K) 44. We assumed that Aurora-A could directly suppress IL-6 mRNA transcription. Unfortunately, the transcription levels of IL-6 were almost identical in the 293T cells transfected with Aurora-A plasmid DNA and the 293T cells transfected with control plasmid DNA (data not shown).

Phosphorylation of STAT1 at Thr749 stabilized IL-6 mRNA and induced an increase in the production of IL-6 45. We therefore assessed the levels of activated STAT1 in CD19^+^ B cells sorted from either the* Aurka^f/f^* mice or the *Aurka^f/f^;Cd19^Cre/+^* mice. We found that the levels of STAT1 were also increased in the *Aurka^f/f^;Cd19^Cre/+^* B cells (Figure 7F), although we were unable to examine p-STAT1 (Thr749) expression due to the lack of a commercial anti-p-STAT1 (Thr749), which was consistent with our unpublished data showing that inhibition of Aurora-A activity or deletion of Aurora-A contributed to epigenetic activation of STAT1. These observations indicated that deletion of Aurora-A in CD19^+^ B cells could promote STAT1 phosphorylation at Thr749, leading to an increase in the levels of IL-6 mRNA, at least partially.

Overall, the current study demonstrated that in CD19^+^ B cells, silencing *Aurka* led to an increase in IL-6 production, promoting STAT3 activation, which in turn contributed to TPO transcription, promoting megakaryocyte differentiation and leading to an increase in platelet number.

## Supplementary Material

Supplementary figures.

## Figures and Tables

**Table 1:** Real time RT-PCR primers.

Gene	Direction	Primer
*Aurka*	Forward	5'-CGTCTTGGTGACTGAGCAGA-3'
	Reverse	5'-GACCTGCTCCAAGTTTCTGG-3'
* Tpo*	Forward	5'-GCTCTTTGCTGGAACCTCAC-3'
	Reverse	5'-AGGCTTGGAGAAGGAGGAAG-3'
*Mpl*	Forward Reverse	5'-TCACCTTGGTGACTGCTCTG-3' 5'-AGCATGCCTCAGTCTCCTGT-3'
*Actb*	Forward	5'-GCTACAGCTTCACCACCACA-3'
	Reverse	5'-TCTCCAGGGAGGAAGAGGAT-3'
*Gata1*	Forward	5'-AGCAACGGCTACTCCACTGT-3'
	Reverse	5'-CCGGTTCTGACCATTCATCT-3'

## Abbreviations

Aurora-A: Aurora kinase A
PB: peripheral blood
TPO: thrombopoietin
BM: bone marrow
STAT3: signal transducer and activator of transcription-3
DSS: dextran sodium sulfate
WT: wild-type
GAS: gamma activated sequence
AMR: Ashwell-Morell receptor
SCF: stem cell factor
mTPO: murine TPO
RP: Reticulated platelets
PRP: platelet rich plasma
DAPI: 4', 6-diamidino-2-phenylindole
vWF: von Willebrand factor
CFU: Colony forming unit
MK: megakaryocyte
ELISA: Enzyme-linked immunosorbent assay
RCB: recirculating B cells
IMB: immature B cells
PPB: pre/pro B cells
MB: mature B
FOB: follicular B
MZB: marginal zone B
WBCs: white blood cells
RBCs: red blood cells
DMs: demarcation membranes
HSCs: hematopoietic stem cells
BEMPs: bipotential erythroid-megakaryocyte progenitors
