# Supplementary material for: Aurka loss in CD19+ B cells promotes megakaryocytopoiesis via IL-6/STAT3 signaling-mediated thrombopoietin production
Source: Theranostics. 2021 Mar 4;11(10):4655–71. doi: 10.7150/thno.49007 (PMC7978311; doi:10.7150/thno.49007)
Supplement: Supplementary file 1 — Supplementary figures. [file thnov11p4655s1.pdf]

Supplementary figure 1

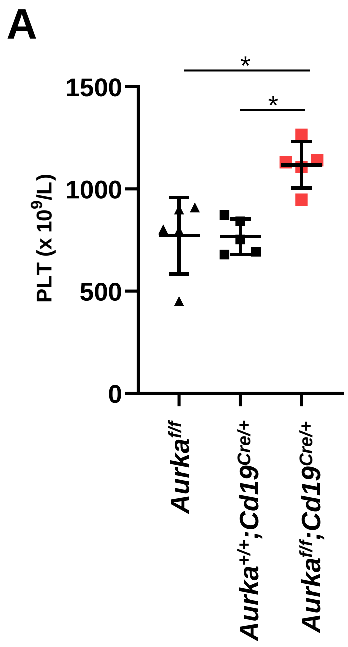

Figure. S1. The total number of platelets in peripheral blood is shown in Figure. 4.

The dot graph shows the total number of platelets in the peripheral blood of the *Aurka*$^{f/f}$ mice, *Aurka*$^{+/+}$*;Cd19*$^{Cre/+}$ mice or *Aurka*$^{f/f}$*;Cd19*$^{Cre/+}$ mice. The graph shows the mean ± SD of one of two independent experiments (n = 5 mice/group) with similar results. Significance was calculated using an unpaired Student's t-test. *, $P < 0.05$.

Supplementary figure 2

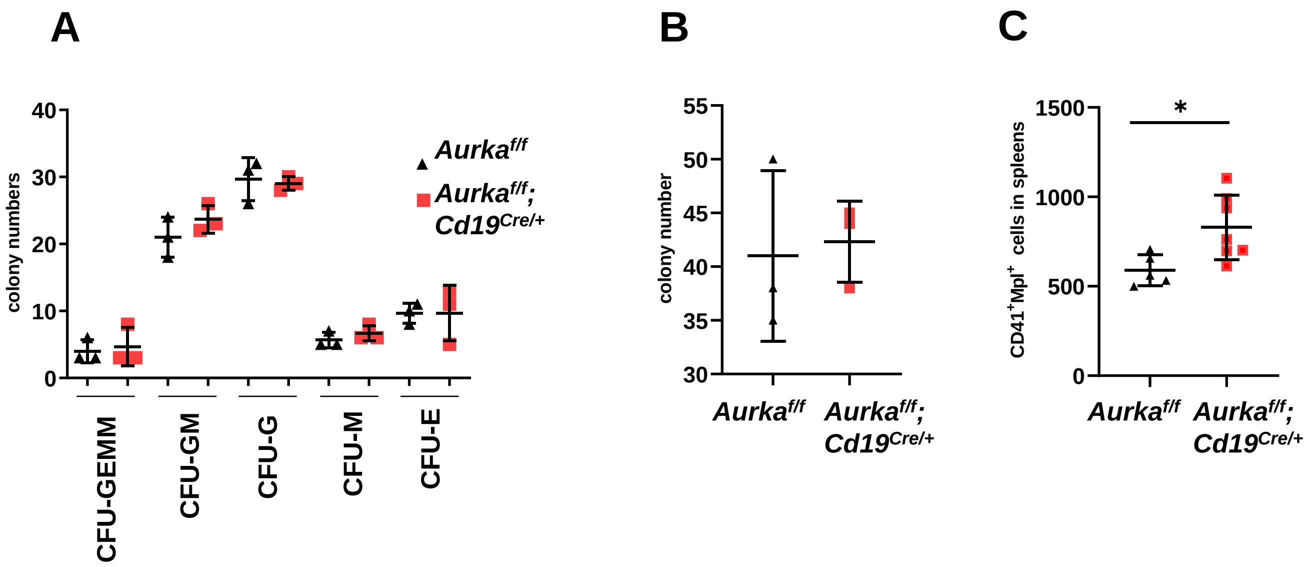

Figure. S2. Deletion of Aurora-A contributed to megakaryocytopoiesis in the spleen without impairing hematopoiesis, as shown in Figure. 5.

(A, B) CFU-GEMM, CFU-E, CFU-GM, CFU-G, CGU-M and CFU-MK colonies from $Lin^{-}c\text{-}Kit^{+}Sca\text{-}1^{-}$ cells in mice with the indicated genotypes (n = 3). The graph shows the mean ± SD of one of two independent experiments with similar results. **(C)** Dot graph showing the number of $CD41^{+}Mpl^{+}$ megakaryocytes in 1×10^4 $c\text{-}Kit^{+}$ splenic nucleated cells removed from the *Aurka*$^{f/f}$ mice (n = 5 mice) or the *Aurka*$^{f/f}$*;Cd19*$^{Cre/+}$ mice (n = 7 mice). The graph shows the mean ± SD of one of two independent experiments with similar results. Significance was calculated using an unpaired Student's t-test. *, $P < 0.05$.

Supplementary figure 3

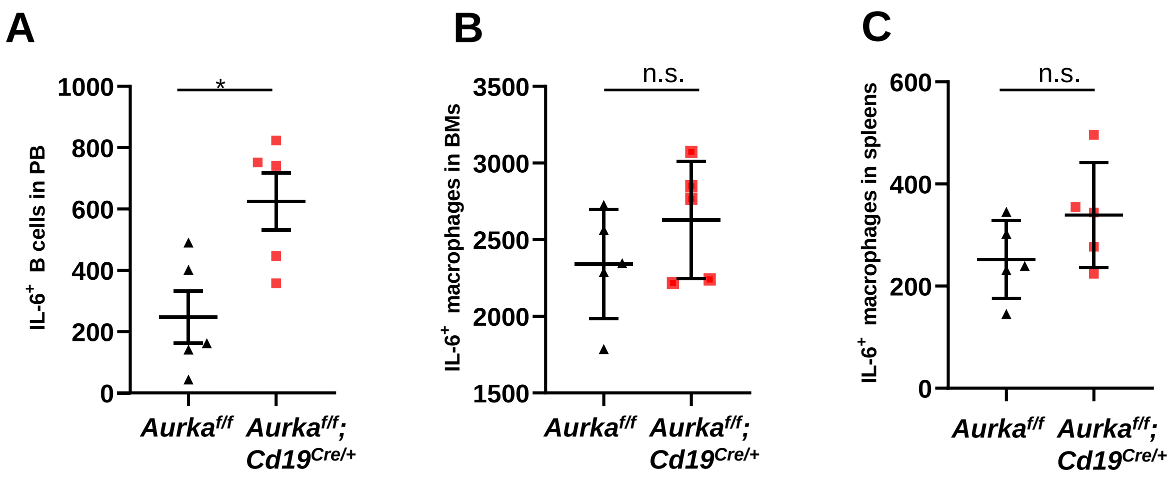

Figure. S3. *Aurka* deletion induced B cells or macrophages expressing IL-6, as shown in Figure. 7.

(A) Dot graph shows the number of $CD19^+IL\text{-}6^+$ B cells in 3×10^4 $CD45^+CD3^-$ nucleated cells from PB of the *Aurka*$^{f/f}$ mice or the *Aurka*$^{f/f}$;*Cd19*$^{Cre/+}$ mice. *, $P < 0.05$. **(B)** Dot graph shows the number of $CD11b^+F4/80^+$ macrophages expressing IL-6 in 5×10^4 $CD45^+$ BM nucleated cells from the *Aurka*$^{f/f}$ mice or the *Aurka*$^{f/f}$;*Cd19*$^{Cre/+}$ mice. n.s., not significant. **(C)** Dot graph shows the number of $CD11b^+F4/80^+$ macrophages expressing IL-6 in 5×10^4 $CD45^+$ splenic nucleated cells from the *Aurka*$^{f/f}$ mice or the *Aurka*$^{f/f}$;*Cd19*$^{Cre/+}$ mice. n.s., not significant. (**A-C**) The graph shows the mean ± SD of one of two independent experiments with similar results. Significance was calculated using an unpaired Student’s t-test.
